# Characteristics of Hospital Acquired Pressure Ulcer and Factors Affecting Its Development: A Retrospective Study

**DOI:** 10.7759/cureus.11992

**Published:** 2020-12-09

**Authors:** Hatan Mortada, Nader Malatani, Basim A Awan, Hattan Aljaaly

**Affiliations:** 1 Division of Plastic Surgery, Department of Surgery, College of Medicine, King Saud University, Riyadh, SAU; 2 Department of Emergency Medicine, King Fahad General Hospital, Jeddah, SAU; 3 Division of Plastic Surgery, Department of Surgery, Faculty of Medicine, King Abdulaziz University, Jeddah, SAU

**Keywords:** pressure injury, hospital acquired, risk factors, characteristics, age, saudi arabia, wound

## Abstract

Background

Worldwide, pressure ulcers (PUs) have been implicated in costing billions annually, with 60,000 deaths out of 2.5 million hospitalized patients resulting from complications related to PU. The prevention of PU reduces the incidence of other illnesses, decreases the financial costs, and improves the quality of life for patients. We aimed to identify the most influential factors that increased the risk of developing PUs among hospitalized patients at a university hospital according to the Waterlow scale.

Methods

Data were collected retrospectively from patients who developed PUs between January 2016 and December 2018 at King Abdulaziz University Hospital, Jeddah, Saudi Arabia, and were evaluated using the Waterlow PU risk assessment tool. The analysis was performed using the Statistical Package for Social Science (SPSS), version 23.0 (IBM, Armonk, NY).

Results

A total of 272 cases were included in this study. The highest number of cases (n = 83, 30.5%) belonged to the age group of 50 to 64 years. The majority of patients had stage 2 PUs (165, 60.7%). The most frequent location of PU was the “back” (97, 35.7%). A history of undergoing major surgery was significantly associated with a higher stage of PU (p = 0.040). The mean Waterlow PU score for all cases was 27.19 ± 13.143. There was a moderate positive correlation between the neurological deficit score and the Waterlow PU score (correlation coefficient: 0.447, p < 0.001). Multinomial logistic regression analysis revealed that increasing age is a significant predictive factor for developing higher stages of PUs (p = 0.046).

Conclusion

Major surgery, neurological deficit, low hemoglobin level, and increasing age were strong predictors for developing higher stages of PU. Therefore, healthcare contributors should consider these risks when applying a comprehensive PU management plan.

## Introduction

Pressure ulcer (PU) is a common medical problem that affects patients in healthcare settings worldwide [1]. A PU is also known as bedsore, pressure injury, pressure sore, or decubitus ulcer, and its defined as damage that is localized to the skin or/and underlying soft tissue, be it linked to a medical device or skin over a bony prominence. Pressure injuries may occur as an intact skin or as an open painful or painless ulcer, resulting from prolonged or/and intense pressure in combination with shear [2]. Across the Middle East, the prevalence of a PU is estimated to be 7-44.4% [3,4]. Previous research has been conducted in a 144-bed governmental hospital in Saudi Arabia, and they have found that the prevalence of hospital-acquired PU was 7.5% [5].

In fact, the prevalence of PU also defers long-term care, acute care, home care, and rehabilitative care by healthcare setting [3]. Patients in the intensive care unit (ICU) have a high risk of developing PU, with an estimated incidence between 3.3% and 52.9% [6,7]. Globally, PU has been implicated in $11 billion in costs annually, and in the United States, 60,000 deaths out of 2.5 million hospitalized patients have resulted from complications related to PU, each year [8]. The cost to establish PU prevention to our patients at risk can tremendously affect the healthcare systems’ resources [9]. Prevention of PU by involving the patient and their families plays a major role in reducing the incidence of other illnesses, decrease the financial costs, and improve the quality of life for our patients [10-12]. Preventing PUs from occurrence is the key principles in its management. The multidisciplinary managing team should not only focus on the wound but also take a broad approach that needs the patient and their family [13]. PU represents an interplay of a combination of factors contributing to its development from both the patient and the environment [14]. According to various prospective studies, factors such as low serum albumin level, age, mobilization, exercise, diabetes intake, and skin PU status have been found to increase the risk of developing PU [15,16]. However, there has been no single factor that can determine the risk of PU development [17]. Therefore, this observational retrospective study aimed to identify and determine the most influential factors that increase the risk of developing PUs among hospitalized patients at a university hospital according to the Waterlow scale.

## Materials and methods

Study design and setting

We performed a retrospective cohort study targeting patients of both sexes who developed PUs between January 2016 and December 2018 at King Abdulaziz University Hospital (KAUH), Jeddah, Saudi Arabia. In order to better reflect our results, we intended to include all patients who experienced hospital-acquired PUs, were over 18 years of age, were not reported to have PUs prior to admission to KAUH, and were assessed using the Waterlow score throughout hospitalization. A list of the medical record of a number of patients who satisfied the inclusion criteria was obtained.

Data collection sheet

After reviewing the literature, we formulated a collection sheet to enter the data based on several published ones [14,17]. The data were collected retrospectively from the Phoenix (KAUH database) using excel sheets composed of 20 variables, including the patients’ demographic characteristics (sex, age, length of stay (LOS), comorbidities, admitted unit), stage and site of the PU, those of the Waterlow PU risk assessment tool (including body mass index (BMI), appetite, mobility, continence, skin type/visual risk areas, special risks/medications, tissue malnutrition, neurological deficit, and major surgery/trauma), and laboratory findings (albumin and hemoglobin levels).

Statistical analysis

Data were checked for errors and completeness. Descriptive statistics were used to present the baseline characteristics and all PU-related variables. Continuous variables were checked for normality using the Kolmogorov-Smirnov test and Shapiro-Wilk test. Feeding route, mobility status, continence, risk of medications, tissue nutrition, major surgery, and comorbidities were presented for all stages of PU, and the relationship of the former variable to the latter was observed by chi-square test. Correlations between all continuous variables (neurological deficit score, LOS, albumin, hemoglobin, and Waterlow PU score) were assessed using Spearman’s rank correlation test. A multinomial logistic regression model was performed by counting the stages of PU as the dependent variable and LOS, albumin level, hemoglobin level, Waterlow PU score, neurological deficit score, sex, comorbidities, and age as independent variables. The analysis was performed using the Statistical Package for Social Science (SPSS), version 23.0 (IBM, Armonk, NY, USA).

Ethics approval and consent to participate

Patient medical records were obtained after participants’ written consent, and the data were collected after we received ethical approval for this study from the Institutional Review Board and the Research Ethics Committee of King Abdulaziz University in Jeddah, Saudi Arabia. (Reference No. 26‐18).

## Results

A total of 272 cases were included in this study, of which 145 (53.3%) were males. The highest number of cases (n = 83, 30.5%) belonged to the age group of 50 to 64 years (Table 1).

**Table 1:** Baseline characteristics of all cases (n = 272). MICU: medical intensive care unit, SICU: surgical intensive care unit, ER: emergency, FMW: female medical ward, MMW: male medical ward, Gyn: gynecology ward, MSW: male surgical ward, BMI: body mass index.

Characteristics	N	%
Unit		
MICU	68	25.0
SICU	51	18.8
ER	41	15.1
FMW	32	11.8
MMW	14	5.1
Gyn	13	4.8
MSW	11	4.0
Others	42	15.4
Gender		
Male	145	53.3
Female	127	46.7
Age group (years)		
14-49	67	24.6
50-64	83	30.5
65-74	63	23.2
75-80	31	11.4
81+	28	10.3
BMI		
Below average (BMI <20)	36	13.2
Average (BMI 20-24.9)	122	44.9
Above average (BMI 25-29.9)	55	20.2
Obese (BMI >30)	59	21.7
Feeding		
Poor	48	17.6
No/anorexia	58	21.3
Nasogastric tube/fluids only	93	34.2
Average	73	26.8
Motility		
Apathetic (sedated/depressed/reluctant to move)	15	5.5
Bedbound (unconscious/unable to change position/traction)	143	52.6
Chair bound (unable to leave the chair without assistance)	12	4.4
Fully mobile	45	16.5
Restless/fidgety	8	2.9
Restricted (restricted by severe pain or disease)	49	18.0
Continence		
Catheterized with fecal incontinence	90	33.1
Complete/catheterized	131	48.2
Urinary and fecal (double) incontinence	43	15.8
Urine incontinence	8	2.9
Major surgery		
No surgery	241	88.6
On table >2 hours (up to 48 hours post-op)	12	4.4
On table >6 hours	13	4.8
Orthopedic below waist/spinal (up to 48 hours post-op)	6	2.2

One hundred sixty-five (60.7%, n = 165) patients had stage 2 PUs, 57 (21%) had stage four, and 45 (16.5%) had stage one. The most frequent location of PU was the “back” (97, 35.7%), followed by the “sacral region” (96, 35.3%). The most common skin type was “dry/itchy” (179, 65.8%). Only 73 (26.8%) cases had “normal/average” food intake while others required a nasogastric tube or parenteral nutrition. More than half of the patients were bedbound (143, 52.6%). Urinary and fecal (double) incontinence was present in eight (2.9%) cases. The clear majority (241, 88.6%) did not undergo surgery.

Table 2 presents the distribution of feeding, mobility, continence, risk of medications, tissue malnutrition, major surgery, and comorbidities according to stages of PU. Major surgery was significantly associated with a higher stage of PU (p = 0.040).

**Table 2:** Distribution of all cases by feeding, mobility, continence, risk of medications, tissue malnutrition, major surgery, and comorbidities by stage of pressure ulcer.

Variables	Stage 1 (%)	Stage 2 (%)	Stage 3 (%)	Stage 4 (%)	p-Value
Feeding					0.196
Poor	15.6	17.0	20.0	21.1	
No/anorexia	11.1	21.2	60.0	26.3	
Nasogastric tube/fluids only	44.4	35.2	20.0	24.6	
Average	28.9	26.7	0.0	28.1	
Mobility					0.526
Fully mobile	17.8	15.8	0.0	19.3	
Not fully mobile	82.2	84.2	100.0	80.7	
Continence					0.941
Catheterized with fecal incontinence	37.8	32.7	40.0	29.8	
Complete/catheterized	37.8	49.7	40.0	52.6	
Urinary and fecal (double) incontinence	20.0	15.2	20.0	14.0	
Urine Incontinence	4.4	2.4	0.0	3.5	
Risk of medications					0.988
Yes	75.6	74.5	80.0	73.7	
No	24.4	25.5	20.0	26.3	
Tissue malnutrition					0.764
No	26.7	21.8	20.0	28.1	
Yes	73.3	78.2	80.0	71.9	
Major surgery					0.040
No	91.1	84.8	100.0	96.5	
Yes	8.9	15.2	0.0	3.5	
Comorbidities					0.073
No	77.8	84.8	80.0	57.9	
Yes	22.2	15.2	20.0	42.1	

The mean Waterlow PU score for all cases was 27.19 ± 13.143. Table 3 shows the correlation between the continuous variables; a moderate uphill correlation between neurological deficit score and Waterlow PU score was observed (correlation coefficient: 0.447, p < 0.001). Multinomial logistic regression analysis revealed that increasing age was a significant predictive factor for developing higher stages of PUs (p = 0.046).

**Table 3:** Correlation between neurological deficit score, LOS in hospital, albumin, hemoglobin, and Waterlow PU score. PU: pressure ulcer, LOS: length of stay.

Correlation matrix	Neurological deficit score	LOS	Albumin	Hemoglobin	Waterlow PU score
Neurological deficit score					
Correlation coefficient	1.000	0.035	−0.100	−0.210	0.447
p-Value	-	0.561	0.123	0.001	0.000
LOS					
Correlation coefficient		1.000	−0.092	−0.125	−0.015
p-value		-	0.156	0.040	0.804
Albumin					
Correlation coefficient			1.000	0.204	−0.121
p-value			-	0.002	0.062
Hemoglobin					
Correlation coefficient				1.000	−0.223
p-value				-	0.000
Waterlow PU score					
Correlation coefficient					1.000
p-Value					-

## Discussion

Overall, 272 patients were involved in this article. The majority of our patients aged from 50 to 64 years (83 cases, 30.5%). Most of them had two pressure injuries (165, 60.7%). The “back” was the most common location of the PU (97, 35.7%). Having a history of undergoing a major surgical procedure was a significant factor associated with a deeper stage of PU (p = 0.04). The overall mean of the Waterlow PU score for all the involved cases was 27.19 ± 13.143. PU can occur in various settings, at home and in any hospital ward or department. In admitted patients, pressure damage has a prevalence of 3-6% [18,19]. Meanwhile, the incidence of developing a PU after surgery is 54.8% [20]. Hence, adequate perception and knowledge regarding PU prevention strategies play a major role in preventing PUs [21]. PU does not only cause a significant economic burden and raise the workload of healthcare providers but also disturbs the patient as it causes pain, and the pain, exudation, and body look disruption have negative effects on the quality of life of the patient and prevent wound healing [22]. Identifying risk factors is the most significant and important method to reduce this burden. Therefore, the goal of this study was to identify and evaluate the most influential factors for the development of PUs among hospital patients according to the Waterlow scale. We found that the mean length of hospital stay was 47 days, but this finding was contradictory to that of Sayar et al. [23]. Moreover, the mean Waterlow PU score for all participants was 27.19 ± 13.143, which suggested very high-PU risk, and this result was consistent with previous studies [23,24]; thus, the Waterlow scoring system was an adequate instrument for risk assessment. The majority of patients included in the study had second-degree PUs (165 patients, 60.7%). However, higher grades and more severe injury to pressure were seen in another study, including grades 3 and 4 [25]. Another important finding was that the most common location of a PU in our article was the “back” (35.7%), followed by the “sacral region” (35.3%), and this was also observed in a study conducted by Sayar et al. [23]. This finding was explained by the fact that for the majority of patients with the head and trunk raised between 15 and 45 degrees, known as the semi-fowler position, PUs are situated on their back. Therefore, understanding the position most vulnerable to the PU can be of great help in preventing PUs. In our research, multinomial logistic regression analysis revealed that increasing age was a major predictive factor for developing higher stages of PU (p = 0.046) when it comes to aging as a risk factor for PU. This was consistent with most previous research [26,27]. Furthermore, over half of our participants were over 50 years old as most older people tend to have less mobility and movement as well as an increased risk of comorbidities [28]. In our sample, the rate of bedbound cases (unconscious/incapable of changing position/traction) was 52.7%. Immobilization has a detrimental impact on the body as a whole, which increases the risk of developing a PU [16]. Statistically significant variations in PU and hemoglobin level were observed. Anemia has been shown to have significant implications in PU [29]; however, another study concluded that low hemoglobin level has no effect on the occurrence of PU [30]. Although this present article has reached its target, there are some important limitations. The study's main limitation was the unreliable, vague, contradictory, and/or incomplete details in the medical records. This could be explained by the lack of continuity that potentially had an indirect effect on follow-up and clinical treatment in the reporting process. Another drawback was that the data did not specify whether the PU was sustained in the hospital or community as well as the exact site of the PU. Moreover, the sample was collected from a single medical institution. Therefore, we suggest prospective studies with a larger sample size to analyze all variables in compliance with the Waterlow parameters and their relationships.

## Conclusions

This retrospective study of patient pressure injury medical records found that major surgery, limited mobility, neurological impairment, low hemoglobin level, inadequate oral nutrition, and older age are factors and good predictors for the occurrence of higher pressure injury levels. According to the results of our single-centered study, healthcare contributors should consider these risks when applying a comprehensive pressure injury management plan.
